# Cross-validation to select Bayesian hierarchical models in phylogenetics

**DOI:** 10.1186/s12862-016-0688-y

**Published:** 2016-05-26

**Authors:** Sebastián Duchêne, David A. Duchêne, Francesca Di Giallonardo, John-Sebastian Eden, Jemma L. Geoghegan, Kathryn E. Holt, Simon Y. W. Ho, Edward C. Holmes

**Affiliations:** Marie Bashir Institute of Infectious Diseases and Biosecurity, Charles Perkins Centre, Sydney Medical School, University of Sydney, Sydney, NSW 2006 Australia; School of Life and Environmental Sciences, University of Sydney, Sydney, NSW 2006 Australia; Department of Biochemistry and Molecular Biology, Bio21 Molecular Science and Biotechnology Institute, The University of Melbourne, Melbourne, VIC 3010 Australia; Centre for Systems Genomics, The University of Melbourne, Melbourne, VIC 3010 Australia

**Keywords:** Model selection, Cross-validation, Bayesian phylogenetics, Molecular clock, Demographic models, Marginal likelihood

## Abstract

**Background:**

Recent developments in Bayesian phylogenetic models have increased the range of inferences that can be drawn from molecular sequence data. Accordingly, model selection has become an important component of phylogenetic analysis. Methods of model selection generally consider the likelihood of the data under the model in question. In the context of Bayesian phylogenetics, the most common approach involves estimating the marginal likelihood, which is typically done by integrating the likelihood across model parameters, weighted by the prior. Although this method is accurate, it is sensitive to the presence of improper priors. We explored an alternative approach based on cross-validation that is widely used in evolutionary analysis. This involves comparing models according to their predictive performance.

**Results:**

We analysed simulated data and a range of viral and bacterial data sets using a cross-validation approach to compare a variety of molecular clock and demographic models. Our results show that cross-validation can be effective in distinguishing between strict- and relaxed-clock models and in identifying demographic models that allow growth in population size over time. In most of our empirical data analyses, the model selected using cross-validation was able to match that selected using marginal-likelihood estimation. The accuracy of cross-validation appears to improve with longer sequence data, particularly when distinguishing between relaxed-clock models.

**Conclusions:**

Cross-validation is a useful method for Bayesian phylogenetic model selection. This method can be readily implemented even when considering complex models where selecting an appropriate prior for all parameters may be difficult.

**Electronic supplementary material:**

The online version of this article (doi:10.1186/s12862-016-0688-y) contains supplementary material, which is available to authorized users.

## Background

Evolutionary analyses of gene sequence data are increasingly reliant on model-based phylogenetic approaches. In recent years, this has been given substantial impetus by the surge in genome-scale data, improvements in computational power, and the application of Bayesian statistical methods to phylogenetics [1]. Statistical models are typically used to describe the substitution process in nucleotide or amino acid sequences [2], diversification and demographic processes [3, 4], and patterns of evolutionary rate variation among lineages [5]. In a Bayesian framework, the various components describing different aspects of the evolutionary process collectively form the hierarchical model.

The accuracy of phylogenetic inference depends on the fit of the Bayesian hierarchical model to the data set being analysed. This includes the extent to which the assumptions of the model are met, and whether the model reasonably describes the data [6]. For example, if a data set sampled from an exponentially growing population is analysed using a model that assumes a constant population size, the estimate of the population size will be highly misleading. Model misspecification can also result in errors in the estimates of other parameters, including the phylogenetic tree and branch lengths [7]. For this reason, model selection forms a critical component of phylogenetic analyses [2].

Likelihood methods for model selection include likelihood-ratio tests and information criteria. The likelihood-ratio test has been widely used in phylogenetics to select substitution models and to test for the strict molecular clock [2]. This method uses the difference in log-likelihoods between two competing models multiplied by 2 as a test statistic. The test statistic follows a *χ*^2^ distribution with degrees of freedom equal to the difference in number of parameters between the two models. A limitation of this approach is that only nested models can be compared. The Akaike Information Criterion (AIC) and Bayesian Information Criterion (BIC) are also popular methods in phylogenetics. Their advantage over the likelihood-ratio test is that it is possible to compare non-nested models. Both of these methods use the maximum likelihood of competing models and penalise the number of parameters to obtain a score. To select a model, the AIC or BIC score is calculated for all the models considered and that with the lowest score is selected [8]. In the case of substitution model selection, the BIC appears to have a better performance than the AIC [9].

Bayesian model selection is usually based on comparison of the marginal likelihoods using Bayes factors [10]. Calculating the marginal likelihood involves integrating the likelihood across parameter values of the model, and weighting by the prior. In phylogenetics, an analytical solution to calculate the marginal likelihood is intractable. Consequently, it is common to use approximate methods of estimating marginal likelihoods, such as importance sampling, path sampling, and generalised stepping-stone sampling [11, 12]. Estimators based on importance sampling, including the harmonic mean and the AICM (a Bayesian analogue to the Akaike Information Criterion), are computationally efficient but unreliable because they have an unacceptably high variance [13–15]. Path-sampling approaches include thermodynamic integration [16] and stepping-stone sampling [17]. Although these estimators are more accurate, they require additional calculations beyond those used to estimate the parameters in the model. To estimate the marginal likelihood, these approaches draw samples from a series of distributions between the posterior and the prior, such that the prior should be carefully selected. In particular, the prior distributions for all parameters should integrate to 1, known as ‘proper priors’ [18].

Conceivably, even when the priors are proper, their arbitrary choice can lead to different models being selected. Although this is well documented in the statistical literature, the effect of the prior in clock model choice remains largely unexplored. A recently developed path-sampling method, known as generalised stepping-stone sampling [11, 12], involves drawing samples from distributions between the posterior and a working distribution, instead of the prior. However, the prior still affects the model selected because it is part of the calculation of the posterior. Importantly, the working distribution for continuous parameters can take a Gaussian shape, but for discrete parameters, such as the genealogy, there are several strategies available to select the working distribution [12].

Lartillot et al. [19] first proposed the use of a cross-validation approach for selecting amino acid substitution models. Its performance was found to be similar to that of Bayes factors using marginal likelihoods. The motivation behind this method is to select models according to their predictive power by splitting the data into ‘training’ and ‘test’ sets. For sequence data, these sets are generated by randomly sampling sites without replacement from the alignment. The training set is used to estimate the parameters of the models being compared. The likelihood of the test set is calculated for each model using the parameter estimates from the training set. The model with the highest likelihood for the test set is regarded as the best-fitting.

In a Bayesian framework, the parameter values are sampled from the posterior distribution obtained from the training set and are used to estimate their likelihood for the test set. The resulting likelihoods are effectively the probability of the test data given the model and parameter estimates under the training set. The model that has the highest mean likelihood for the test set is then regarded as providing the best fit. Because the likelihood of the models is evaluated using a data set that has not been observed (i.e., the test set), artefacts due to over-parameterization are alleviated [19]. Thus, it is not necessary to penalize explicitly for excessive parameters, as in the case of information criteria [8]. To reduce sampling error, the cross-validation procedure can be repeated a number of times, with the likelihood for each model averaged over replicates.

We extend the cross-validation method proposed by Lartillot et al. [19] for substitution models to other components of the Bayesian hierarchical model: the molecular clock model and the demographic model. We also test whether the performance of the method depends on the length of the sequence alignment, because the probability of identifying the optimal model should improve with the amount of data (i.e., statistical consistency).

## Methods

### Cross-validation implementation

In our implementation of cross-validation, we randomly sample half of the sequence alignment without replacement. One half is the training set and the other is the test set, such that the two sets have no overlapping sites. We then analyse the training set using the Bayesian Markov chain Monte Carlo method in BEAST v2.3 [20]. This program requires the specification of a clock model as well as a demographic or speciation model. It estimates the posterior distribution of parameters in the model, including rooted phylogenetic trees with branch lengths in units of time (known as chronograms). We draw samples from the posterior and use P4 v1.1 [21] to calculate the phylogenetic likelihood of the test set given these samples. However, to calculate the phylogenetic likelihood it is necessary to use phylograms (i.e., phylogenetic trees with branch lengths in substitutions per site). We convert the chronograms into phylograms by multiplying branch lengths (in time units) and substitution rates. We draw 1,000 samples from the posterior estimates of the training set, then use each set of sampled parameters to calculate the mean phylogenetic likelihood for the test set. The mean likelihood is compared for different models, and we consider the best model to be that with the highest mean likelihood for the test set. The computer code to conduct our analyses is available online (github.com/sebastianduchene/cv_model_selection).

### Simulations

We used a simulation approach to test the accuracy of cross-validation in selecting clock and demographic models. We considered three molecular clock models; the strict clock (SC), the relaxed uncorrelated lognormal (UCLN) clock, and the relaxed uncorrelated exponential (UCED) clock. First, we sampled from the prior within the BEAST framework to generate ten phylogenetic trees, each with 50 taxa and a root node age of 100 years. The tree topology and relative ages of internal nodes were based on a constant-size demographic model. We simulated branch rates according to the three clock models using NELSI v1.0 [22]. For the SC model, we used a rate of 10^−3^ substitutions/site/year, which broadly reflects the substitution rates that have been estimated in a range of RNA viruses, such as HIV [23] and *Dengue virus* [24]. For the UCLN and UCED models, we used a mean of 10^−3^ substitutions/site/year and a standard deviation of 10 % of the mean for the UCLN (note that in the UCED, the mean equals the standard deviation). We then used Pyvolve [25] to simulate the evolution of sequences of length 5,000, 10,000, and 15,000 nt under the Jukes-Cantor substitution model.

We compared all three clock models using the cross-validation method described above, with test and training sets of 50 % of the alignment length. For the BEAST analysis, we used a chain length of 10^7^ steps, with samples drawn every 5,000 steps, and discarding 10 % of the chain as burn-in. Whenever the effective sample size for any of the parameters was below 200, we doubled the chain length and halved the sampling frequency.

We also conducted simulations using two different demographic models: the constant-size coalescent (CSC) and the exponential-growth coalescent (EGC). We obtained trees in BEAST by sampling chronograms from the prior under the two demographic models. For the EGC model, we set the growth rate to 0.25, which is similar to that observed in some viruses [26]. We used the strict-clock model with a rate of 10^−3^ substitutions/site/year and simulated sequence evolution as described above.

### Analyses of empirical data

We analysed four nucleotide sequence data sets to illustrate the performance of cross-validation (i) Enterovirus A71 (EV-A71), with 34 partial polyprotein sequences of 858 nt sampled between 2011 and 2013 (including the day and month) [27]; (ii) West Nile Virus (WNV), with 68 complete sequences of 10,299 nt sampled between 1999 and 2003 [28]; (iii) Rabbit Hemorrhagic Disease Virus (RHDV), with 72 capsid gene sequences of 1,726 nt sampled between 1995 and 2014 [26]; and (iv) whole-genome single-nucleotide polymorphisms of the bacterium *Shigella sonnei,* with a total of 161 sequences of 1,626 nt sampled between 1995 and 2010 [29] (Table 1). The alignments are available online (github.com/sebastianduchene/cv_model_selection).

**Table 1:** Details of four viral and bacterial data sets analysed in this study

Data set	Number of sequences	Alignment length (bp)	Variable sites	Sampling time span	Reference
EV-A71	34	859	101	2011– 2013	[27]
WNV	68	10299	366	1999 – 2013	[28]
RHDV	72	1737	571	1995 – 2014	[26]
*Shigella sonnei*	161	1626	1626	1995 – 2014	[29]

We used the cross-validation method to compare four combinations of clock model and demographic model: SC + CSC, SC + EGC, UCLN + CSC, and UCLN + EGC. These analyses were conducted in BEAST using the same settings as in our analyses of simulated data. We used the GTR + Γ substitution model, accounting for rate heterogeneity among sites which is expected in the empirical data. The sampling times of the sequences were used to calibrate the clock; the four data sets have previously been found to have sufficient temporal structure according to the date-randomization test [30, 31]. For each analysis, we performed ten replicates of the cross-validation procedure, which appears to be sufficient in empirical studies [32]. We conducted two sets of analyses, in which we specified the training set as either 50 or 80 % of the alignment length. We used the mean likelihood across the ten replicates to select the optimal hierarchical model. For comparison, we estimated marginal likelihoods for these model combinations using stepping-stone sampling in BEAST [17].

To investigate potential differences between the models selected using marginal likelihoods and cross-validation, we analysed the complete data sets using UCLN + EGC. Under this model combination, it is possible to obtain a measure of clock-like behavior (the coefficient of variation of branch rates) [33] and the population growth rate. The data display clock-like behavior if the mode of the posterior is close to zero, and a constant population size if the 95 % credible interval of the growth rate includes zero.

## Results

### Simulations

The cross-validation methods yielded mixed results when attempting to distinguish between data generated under the strict- and the relaxed-clock models. In all simulations under the SC model, cross-validation correctly identified the model used to generate the data (Table 2). Similarly, the SC model had no support when the data were generated under either of the relaxed-clock models. This result did not depend on sequence length, suggesting that even the smaller data sets, of 5,000 nt and a training set of 2,500 nt, were sufficiently informative. In contrast, distinguishing between the UCLN and the UCED models was more difficult. The UCLN model had the strongest support for most of the data sets, even for those generated under the UCED model. Interestingly, an increase in sequence length led to a greater frequency of the UCED model being correctly chosen for data generated under the same model. For the simulations performed using the UCED model and with an alignment length of 5,000, 10,000, and 15,000 nt, the UCED model was selected with a frequency of 0.1, 0.4, and 0.6, respectively. Notably, in a previous study using marginal likelihood, an alignment of 2,500 nt from 32 taxa was sufficient to distinguish between relaxed-clock models [18].

**Table 2:** Molecular-clock models selected for data sets simulated with three different sequence lengths (nt) and using three different clock models: the strict clock (SC), uncorrelated lognormal relaxed clock (UCLN), uncorrelated exponential relaxed clock (UCED)

Clock model used for simulation	Clock model used for analysis								
	5,000 nt			10,000 nt			15,000 nt		
	SC	UCLN	UCED	SC	UCLN	UCED	SC	UCLN	UCED
SC	1.00	0.00	0.00	1.00	0.00	0.00	1.00	0.00	0.00
UCLN	0.00	0.80	0.20	0.00	0.60	0.40	0.00	0.80	0.20
UCED	0.00	0.90	0.10	0.00	0.60	0.40	0.00	0.40	0.60

The numbers indicate the frequency with which each model was selected, out of ten simulation replicates

For the simulations under different demographic models, the cross-validation method incorrectly supported the EGC model for the data generated under the CSC, with a frequency of 0.3 for alignments of 5,000 nt, and 0.4 for those of 10,000 and 15,000 nt. The method was much better at identifying the EGC model, which was supported with a frequency of 0.9 for all sequence lengths (Table 3). This result indicates that under these simulation conditions, cross-validation is more efficient at detecting under- than over-parameterization for demographic models. Similar results have been reported in the context of clock-model adequacy [34].

**Table 3:** Demographic models selected for replicate data sets simulated with three different sequence lengths (nt) and using two different demographic models: the constant-size coalescent (CSC) and exponential-growth coalescent (EGC), with a growth rate of 0.25

Demographic model used for simulation	Demographic model used for analysis					
	5,000 nt		10,000 nt		15,000 nt	
	CSC	EGC	CSC	EGC	CSC	EGC
CSC	0.70	0.30	0.40	0.60	0.40	0.60
EGC	0.10	0.90	0.10	0.90	0.10	0.90

Each row corresponds to simulations performed using one of the two demographic models

### Empirical data

In our analyses of empirical data, we found that the SC + EGC model was supported for EV-A71 and WNV, using either 50 or 80 % of the data for the training set (Table 4). This result is consistent with the findings of the studies that originally analysed these data sets using marginal likelihoods [27, 28]. For EV-A71, the model selected using cross-validation matched that chosen by comparison of marginal likelihoods. For WNV, however, UCLN + EGC had the highest marginal likelihood. The UCLN + EGC model had the highest support for the RHDV data set according to both cross-validation and marginal likelihoods, which is again consistent with the findings of the original study [26]. For *Shigella sonnei*, cross-validation supported the SC + CSC model, whereas marginal likelihoods supported the UCLN + EGC model. There was some overlap in the standard errors of the mean likelihoods in some of the model comparisons, suggesting relatively weak support for the model with the greatest statistical fit. For example, in WNV using a training set of 80 % of the sites, the two models with highest statistical fit, SC + CSC and SC + EGC, had likelihoods of −7698.8 and −7699.5, with standard errors of 0.4 and 0.3, respectively (Table 4). This result underscores the importance of conducting a large number of cross-validation replicates, particularly when assessing demographic models.

**Table 4:** Comparison of molecular clock and demographic models for four empirical data sets: Enterovirus A71 (EV-A71), West Nile Virus (WNV), Rabbit Hemorrhagic Disease Virus (RHDV), and *Shigella sonnei*

Method	Data set	SC + CSC	SC + EGC	UCLN + CSC	UCLN + EGC
Cross validation (50 % training; 50 % test)	EV-A71	−1129.4(±3.1)	**−1122.3(±2.0)**	−1921.9(±9.8)	−1396.1(±12.0)
	WNV	−8216.7(±1.3)	**−8213.1(±2.5)**	−8648.9(±5.3)	−8691.3(±5.0)
	RHDV	−6456.1(±0.6)	−6908.8(±0.3)	−6102.8(±1.3)	**−6101.9(±1.6)**
	*Shigella sonnei*	**−7698.8(±0.4)**	−7699.5(±0.3)	−25997.4(±7.9)	−25630.9(±6.3)
Cross validation (80 % training; 20 % test)	EV-A71	−443.0(±1.8)	**−440.8(±1.0)**	−1246.5(±4.7)	−1286.7(±14.8)
	WNV	−3615.2(±2.6)	**−3614.9(±2.5)**	−3900.0(±19.9)	−3857.1(±19.2)
	RHDV	−2394.7(±0.6)	−2393.5(±0.7)	−2336.7(±1.0)	**−2279.2(±0.8)**
	*Shigella sonnei*	**−2978.2(±1.9)**	−2979.8(±2.0)	−3172.3(±11.6)	−3032.5(±10.0)
Marginal likelihoods using stepping stone	EV-A71	−2017.0	**−2014.7**	−2017.9	−2078.6
	WNV	−18012.7	−17998.2	−18009.2	**−17991.4**
	RHDV	−11323.8	−11292.6	−11271.5	**−11245.8**
	*Shigella sonnei*	−14739.6	−14746.5	−14717.8	**−14717.8**

The models correspond to four combinations of clock and demographic models: strict clock (SC), uncorrelated lognormal clock (UCLN), constant-size coalescent (CSC), and exponential-growth coalescent (EGC). Mean log likelihoods across ten replicates are given for the test set from each data set, using training sets of 50 and 80 % of the total alignment length. Marginal log likelihoods using stepping-stone sampling are also shown for comparison. Values in bold correspond to the highest log likelihood in each case. Values in parentheses indicate the standard error around the mean likelihood for ten cross-validation replicates

**Fig. 1:**
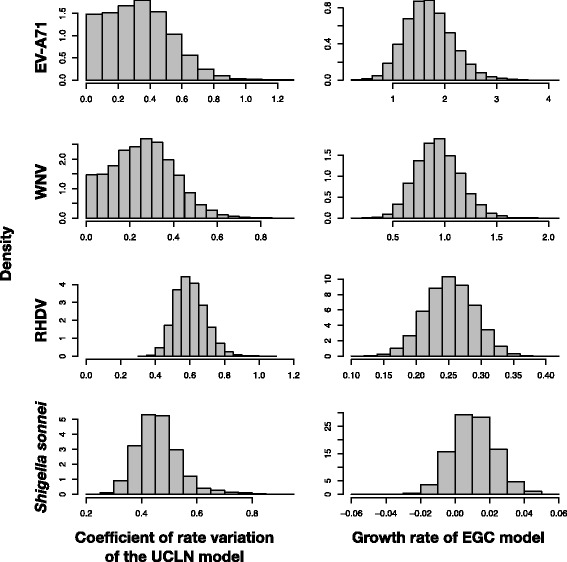
Posterior distributions of the coefficient of variation of branch rates and the population growth rate for four empirical data sets: Enterovirus A71 (EV-A71), West Nile Virus (WNV), Rabbit Hemorrhagic Disease Virus (RHDV), and *Shigella sonnei*. Estimates were made using the uncorrelated lognormal clock (UCLN) and the exponential-growth coalescent (EGC). A coefficient of variation of branch rates that approaches zero indicates that evolution has been clock-like. A growth rate including zero indicates that population size has been constant

Our analyses under the UCLN + ECG demonstrated that EV-A71 and WNV display clock-like behavior, whereas RHDV and *Shigella sonnei* have substantial rate variation among lineages. All of the virus data sets displayed evidence of population growth, whereas *Shigella sonnei* appeared to have had a constant population size (Fig. 1). This indicates that neither model-selection method has a specific bias towards or against parameter-rich models, at least in these data sets.

## Discussion

The cross-validation method was effective in detecting rate variation among lineages. However, distinguishing between different relaxed-clock models was more difficult. Out of the two relaxed-clock models being compared, the UCLN was selected in most cases. For the data simulated under the UCED model, increasing sequence length appeared to increase the frequency with which the UCED was selected. However, our longest sequence alignments contained 15,000 nt, such that even large amounts of data might be insufficient to distinguish between the UCLN and UCED models. Although previous studies have also described the difficulties in distinguishing between relaxed-clock models [22, 35], in most cases marginal-likelihood estimation using stepping-stone sampling and Bayesian model averaging proved accurate [13, 18]. In practice, however, the UCED has a mode at zero, such that it may be unsuitable for most data sets. An additional factor that might warrant further study is whether using data sets with many taxa improves clock model selection [13].

Our simulations demonstrated that cross-validation could detect population size growth over time. However, for data generated under a constant population size, it often selected an exponential growth model, a problem that was not alleviated by using longer sequence data. In contrast, previous studies suggest that using marginal likelihoods is more efficient at detecting both constant and growing population sizes [12, 18]. One potential reason for this result is that marginal-likelihood methods are more effective than cross-validation at penalising excessive parameters. However, the EGC model has one parameter more (the growth rate) than the CSC. If the estimate for this parameter has a mode at, or near, zero, then the inferences from the EGC model might be indistinguishable from those using CSC. Under these circumstances, cross-validation selects these models with similar frequency.

Marginal likelihoods and cross-validation selected the same models for two of our four empirical data sets. However, for WNV and for *Shigella sonnei* the two methods selected different models. In both of these data sets, we found very large differences in mean likelihoods using cross-validation for the different models, especially when comparing the UCLN + CSC and the UCLN + EGC with either SC + SCS or SC + EGC (Table 3). Importantly, these differences in likelihoods depended on the size of the training set. As an example, for *Shigella sonnei*, the mean log-likelihoods for SC + SCS and SC + EGC with a training set of 50 % were thousands of log-likelihood units higher than those for UCLN + SCS and UCLN + EGC. In contrast, using a training set size of 80 % resulted in log-likelihood differences of 100 log-likelihood units or less between these models. This might occur because a training set of 50 % in these data is not sufficiently informative to estimate the parameters in the more complex models. For this reason, the size of the training set should be selected according to the complexity of the model. If the training set is very small, it will be difficult to estimate a large number of parameters, leading to excessive penalisation for parameter-rich models. In empirical studies it might be helpful to explore different sizes for the test and training sets to ensure that the results are statistically consistent. For example, if the size of the training set is very small, the likelihood of the test set will be extremely low for complex models and with very high variation among replicates. In such a case, increasing the size of the training set might be beneficial. Finally, an important consideration of marginal likelihood methods is the additional computational time required. In our empirical data analyses we found that most marginal likelihood estimates required only ten more hours of computational time than our cross-validation replicates with 80 % of the sites (Additional file 1: Table: S1). However, the recently developed generalized stepping-stone method has been shown to yield accurate marginal likelihood estimates in a more timely fashion [11, 12].

## Conclusions

Our analyses of simulated and empirical data show that cross-validation provides a useful model-selection method for Bayesian phylogenetics. Although marginal-likelihood methods are more effective in many cases, one potential advantage of cross-validation is that as long as the data are sufficiently informative model choice is not affected by the prior, such that it might be more readily applied than complex hierarchical Bayesian models where selecting appropriate priors for all parameters is difficult. Further research into cross-validation methods has the potential to improve the reliability of model selection in Bayesian phylogenetics.

## Abbreviations

SC, strict clock; UCLN, uncorrelated lognormal clock; UCED, uncorrelated exponential clock; CSC, constant-size coalescent; EGC, exponential growth coalescent; AIC, Akaike information criterion; BIC, Bayesian information criterion

## Additional files

Additional files 1: Table S1. Spreadsheet with computation times in hours for all analyses. For cross-validation analyses we report the mean value over ten cross-validation replicates. For marginal-likelihood calculations we report the computation time of a single analysis. (XLSX 37 kb)
